# Calcium Homeostasis Machinery in the Human Uterus—A Potential Therapeutic Target in Endometrial Cancer

**DOI:** 10.3390/ijms262110253

**Published:** 2025-10-22

**Authors:** Piotr K. Zakrzewski

**Affiliations:** Department of Molecular Neurochemistry, Faculty of Health Sciences, Medical University of Lodz, 92-215 Lodz, Poland; piotr.zakrzewski@umed.lodz.pl

**Keywords:** endometrial cancer, calcium, novel therapies

## Abstract

Endometrial cancer is one of the most common malignancies of the female reproductive system, with incidence rising globally due to population ageing and life-style-related risk factors. Calcium (Ca^2+^) is a ubiquitous second messenger regulating diverse physiological processes, and its dysregulation has been increasingly implicated in carcinogenesis, including endometrial. Altered expression and function of Ca^2+^ channels, pumps, exchangers, and binding proteins disrupt the finely tuned balance of Ca^2+^ influx, efflux, and intracellular storage, leading to aberrant signalling that promotes tumour proliferation, migration, survival, and metastasis. This review summarises current knowledge on the molecular “Ca^2+^ toolkit” in the human uterus, highlighting the role of voltage-gated calcium channels (VGCCs), transient receptor potential (TRP) channels, store-operated calcium entry (SOCE) components, Na^+^/Ca^2+^ exchangers, purinergic receptors, P-type ATPases (SERCA, SPCA, PMCA), ryanodine (RyR) and inositol 1,4,5-trisphosphate (IP_3_R) receptors, and mitochondrial Ca^2+^ uniporter (MCU) complexes in endometrial cancer progression. Multiple Ca^2+^-handling proteins, including CACNA1D, CACNA2D1, TRPV4, TRPV1, TRPM4, MCU, and RyR1, exhibit cancer-associated overexpression or functional changes, correlating with poor prognosis and aggressive disease features. Emerging evidence supports the therapeutic potential of targeting Ca^2+^ homeostasis using small-molecule inhibitors, ion channel modulators or gene-silencing strategies. These interventions may restore Ca^2+^ balance, induce apoptosis or autophagy, and suppress metastatic behaviour. While no clinical trials have yet explicitly focused on Ca^2+^ modulation in endometrial cancer, the diversity of dysregulated Ca^2+^ pathways offers a rich landscape for novel therapeutic strategies. Targeting key components of the Ca^2+^ signalling network holds promise for improving outcomes in endometrial cancer.

## 1. Introduction

Endometrial cancer ranks among the most frequently diagnosed malignancies of the female reproductive system. Based on GLOBOCAN 2022 [1], approximately 420,368 new cases are reported globally each year, resulting in nearly 97,723 deaths. The worldwide incidence rate is approximately 8.4 per 100,000 women [https://gco.iarc.who.int/today, accessed on 28 July 2025]. The higher prevalence of this cancer in developed nations is primarily attributed to lifestyle-related risk factors. Furthermore, the incidence is anticipated to rise in the coming years, mainly due to the ageing of the global population, since this cancer predominantly affects women during and after menopause [2,3].

Clinically, endometrial cancer is divided into two main categories: type I (endometrioid) and type II (non-endometrioid), each with distinct pathological characteristics and aetiologies. Type I accounts for the majority of diagnoses (75–90%) and originates from the glandular epithelium of the endometrial lining. These tumours are typically oestrogen-dependent, low-grade, and associated with relatively favourable outcomes. Most type I cancers are endometrial adenocarcinomas. In contrast, type II tumours, which include papillary serous and clear cell carcinomas, are less common but exhibit more aggressive behaviour and poorer prognoses. Type II endometrial cancer is a non-oestrogen-dependent tumour type [4,5]. Around 75% of cases are diagnosed at an early stage—FIGO stages I and II—where the 5-year overall survival ranges from 75% to 95%. However, in advanced disease (FIGO stages III and IV), survival significantly declines, with 5-year rates dropping to 50–68% and 27–36%, respectively [6].

Oestrogen plays a pivotal role in modulating calcium dynamics within the endometrium by inducing rapid Ca^2+^ mobilisation and altering the expression of calcium-regulatory proteins [7,8,9]. Acute oestrogen stimulation enhances calcium influx, resulting in a transient rise in intracellular Ca^2+^ that activates signalling cascades characteristic of non-genomic pathways. Disruption of this mechanism may facilitate malignant transformation and tumour progression. The immediate calcium surge frequently initiates phosphorylation events in kinase networks and modulates the activity of transcription factors, thereby influencing a range of cellular functions. In contrast, prolonged oestrogen exposure induces lasting alterations in calcium-associated proteins in both normal endometrial epithelial cells (EECs) and cancerous cells [9,10]. Persistently elevated Ca^2+^ levels affect not only epithelial cell behaviour but also the surrounding microenvironment [11,12]. Nonetheless, it remains unclear whether the rapid, non-genomic effects and the long-term regulatory changes in calcium signalling act synergistically in the initiation and progression of endometrial cancer.

Calcium is one of the most essential elements necessary for the proper functioning of the human body. The vast majority of calcium is deposited in the bones and teeth (app. 99%) as a hydroxyapatite compound, while the rest of the calcium circulates in body fluids (app. 1%) in an ionised form (Ca^2+^) [13]. In the context of the entire body, calcium is involved in muscle contraction, participates in blood clotting, fertilisation process, enzyme activation, and hormone secretion, and is the primary building component of the skeleton. At the cellular level, Ca^2+^ is a universal secondary messenger involved in various signalling pathways, the secretion of neurotransmitters, and also plays a role in cell cycle control, gene transcription, cell motility, apoptosis, and autophagy [14,15]. The concentration of calcium in the human body is maintained at a well-defined level by several special calcium transporters, pumps, and ion channels. Intracellular calcium concentration is 10,000-fold lower than its extracellular level. Every minimal alteration in this delicate balance can lead to cell dysfunction and, consequently, result in destabilisation and even cell death [16]. The maintenance of calcium concentration is a dynamic spatiotemporal process involving either calcium release from extracellular reservoirs (app. 1–2 mM) to the cytosol (app. 100 nM) or calcium efflux from both the intracellular organelles (app. 10 µM) to the cytosol and from the cytosol to the extracellular matrix [17]. The millimolar Ca^2+^ gradient across the cell membrane drives calcium influx when Ca^2+^-permeable channels open in response to an adequate stimulus [18]. In response to calcium-mediated cell stimulation, the intracellular calcium concentration increases more than 2-fold, reaching the micromolar level. Moreover, calcium signals may be shaped into the form of oscillations, spikes, and waves, among which the latter can possess a transient or sustained character [16,17]. The maintenance of calcium homeostasis demands highly integrated mechanisms involving feedback loops that are under the control of hormones. Optimal calcium concentration is maintained by a variety of molecules, including Ca^2+^ exchangers, channels, pumps, transporters, and Ca^2+^-binding/buffering proteins, which cooperate to govern rapid changes in cellular Ca^2+^ necessary for calcium-dependent signalling [19]. All proteins involved in the regulation of intracellular calcium concentration provide cells with a calcium signalling “toolkit”. As reported in numerous studies, calcium dyshomeostasis has been observed in various human pathologies, particularly cancer [13,20,21,22,23,24]. Therefore, this calcium “toolkit” can be regarded as a promising target for novel anti-cancer strategies [16].

Focusing on the role of Ca^2+^ in the etiopathology of endometrial cancer reveals that patients have an increased level of this ion in their serum compared to healthy individuals. As demonstrated, an elevated level of ionised calcium in serum correlates with cancer progression, as described in the FIGO classification (FIGO II+IV vs. FIGO I), histopathological grading (G2/3 vs. G1), local nodes metastasis, and positive peritoneal cytology [25]. Moreover, postmenopausal women with diagnosed endometrial cancer display significantly higher total serum calcium (*p* = 0.002) and albumin-corrected serum calcium (*p* = 0.012) than premenopausal patients. The analysis of serum calcium level in the context of the histological origin of endometrial cancer revealed that higher calcium concentration is characteristic of endometrioid cancers in comparison to non-endometrioid type [26].

The disruption of calcium signalling is responsible for cell reprogramming, which leads to the acquisition of a malignant phenotype. Altered calcium signalling contributes to increased cell proliferation and enables cells to avoid programmed cell death. Furthermore, cells become able to escape immunosurveillance effectively, and due to pronounced tumour neovascularisation and increased cell motility, can invade new niches, ultimately leading to the establishment of metastases [27].

In endometrial cancer, abnormal calcium signalling significantly disrupts apoptosis and proliferation pathways, with strong clinical and mechanistic evidence supporting its role in tumour progression. Mechanistically, calcium homeostasis in the human endometrium is oestrogen-dependent. Oestrogen exerts a hierarchical regulatory influence over calcium homeostasis in endometrial cancer through both genomic and non-genomic pathways. Upon activation of classical nuclear receptors, i.e., oestrogen receptor α (ERα) and oestrogen receptor β (ERβ), or non-canonical G protein-coupled oestrogen receptor (GPER), a cascade of calcium-dependent remodelling events is initiated [28]. Calcium-related proteins are upregulated by oestrogen and regulate both rapid calcium increases and long-term changes in protein expression involved in cancer initiation and progression [29]. Additionally, calcium signalling plays critical roles in tumour microenvironment processes and in the acquisition of multidrug resistance [16]. Several key signalling pathways are disrupted by calcium homeostasis deregulation in endometrial cancer, including cytoskeletal and migration pathways, mitochondrial and metabolic processes, signalling related to lysosomal activity, kinase cascade pathways, and apoptotic and proliferative signalling [25,29,30]. These alterations reshape intracellular calcium oscillations and amplitude, which in turn activate key downstream transduction routes, including MAPK (ERK, p38, JNK), and calcineurin/NFAT, thereby promoting cancer cell proliferation, migration, and survival (Chen et al., 2019; Cui et al., 2017; Okomura et al., 2024) [27,31,32]. Calcium influx activates the RhoA/ROCK1 pathway, leading to LIMK/cofilin activation and affecting F-actin and paxillin levels, ultimately promoting metastasis through cytoskeleton remodelling [25]. Oestrogen-induced calcium influx disrupts mitochondrial-related pathways, specifically targeting mitochondrial ROS production and affecting cellular metabolism. Proteomic analysis revealed that calcium influx is involved in the autophagy pathway, as it impacts lysosomal function and membrane integrity, thereby affecting cellular degradation processes [30]. Furthermore, oestrogen-evoked calcium increases the activation of subsequent kinase cascades, affecting both rapid signalling responses and long-term changes in protein expression [29]. Calcium signalling also regulates cell proliferation, apoptosis, and migration through multiple mechanisms, and calcium channel blockers have been shown to modulate these processes. The calcium-apoptosis connection involves mitochondrial-centric mechanisms and interactions with pro-apoptotic factors [33].

Altered expression or abnormal inactivation of Ca^2+^ exchangers, channels, pumps or transporters underlie the disruption of calcium signalling in cancer cells. The review aims to present the current state of knowledge concerning the role of deregulation of calcium homeostasis in pathologies of human endometrium, in particular endometrial cancer development and progression, with an emphasis on the identification of calcium-related targets and putative calcium modulators, blockers, or inhibitors as novel anti-cancer therapeutics.

## 2. Calcium Homeostasis “Toolkit”

**Figure 1:**
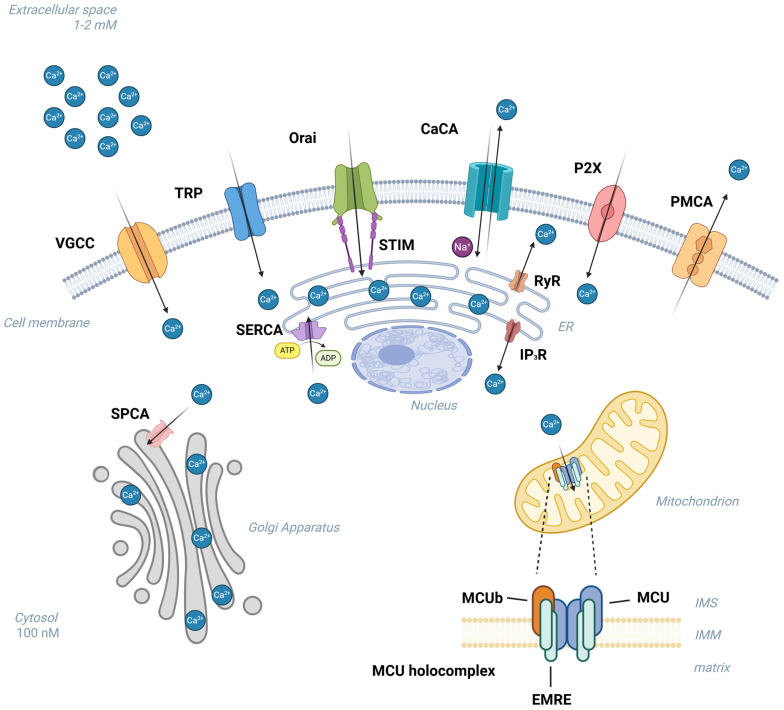
Calcium (Ca^2+^) homeostasis machinery in the human uterus. Different Ca^2+^ transporters, channels, exchangers, and pumps mediate cellular and compartments Ca^2+^ concentration. In the cell membrane, voltage-gated Ca^2+^ channels (VGCC), transient receptor potential channels (TRP), Orai, calcium channel antiporters (CaCA), purinergic P2X receptors, plasma membrane Ca^2+^-ATPases (PMCA) pump, and regulate the transport of Ca^2+^ ions through the cell membrane. Inositol 1,4,5-triphosphate receptors (IP3R), ryanodine receptors (RyR) and sarcoendoplasmic reticulum Ca^2+^-ATPase (SERCA) pump regulate the sequestration of Ca^2+^ in the endoplasmic reticulum. Mitochondrial Ca^2+^ uniporter (MCU) is crucial for controlling the mitochondrial Ca^2+^ uptake. ER—endoplasmic reticulum; EMRE—essential MCU regulator; MCUb—MCU dominant negative subunit beta; IMS—intermembrane space; IMM—inner mitochondrial membrane.

Calcium influx channels/pumps/exchangers are grouped according to their genetic origin and functional properties. The primary classification is based on the destination of transported calcium ions: (1) calcium influx to the cytosol from extracellular space; (2) calcium efflux to the extracellular matrix; (3) bidirectional calcium shuttling between intracellular calcium reservoirs and cytosol. Calcium ions enter the cytoplasm from extracellular reservoirs via voltage-gated Ca^2+^ channel (VGCC) [34], transient receptor potential channel (TRP) [35], Ca^2+^ release-activated Ca^2+^ channel (CRAC) [36], Na^+^/Ca^2+^ exchanger (NCX) and purinergic receptor [37]. IP_3_Rs or ryanodine receptors (RyRs) releases Ca^2+^ from endoplasmic/sarcoplasmic reticulum (ER/SR), whereas Ca^2+^-ATPases are responsible for cytosolic Ca^2+^ translocation to the extracellular space, ER/SR or Golgi apparatus [38,39]. Mitochondrial Ca^2+^ uniporter (MCU) mediates mitochondrial Ca^2+^ reuptake from the cytosol [40]. Figure 1 shows the cellular components involved in maintaining calcium homeostasis of the human uterus.

An essential role in calcium homeostasis can be attributed to molecules, which possess an ability to modulate cellular Ca^2+^ concentration, protecting cells from cytotoxic Ca^2+^ overload, or to decode Ca^2+^ oscillations to control signalling pathways, and in general, are called calcium sensors [41]. These molecules are gathered into a large heterogeneous family of calcium-binding proteins (CBP). Among the members of CBPs can be distinguished intracellular CBPs with or without EF-hand domains, as well as other extracellular CBPs [42]. The CBPs with EF-hand domains are classified based on the presence of two alpha helices oriented perpendicular to each other and accommodating Ca^2+^ or Mg^2+^ at an angle between helices [43]. The CBPs with EF-hand motif include distinct protein families, such as parvalbumin [44], S100 [45], calmodulin [46], calcineurin [47], neuronal Ca^2+^ sensor (NCS) proteins [48], and calcium and integrin-binding protein 1 (CIB1) [49], which contains four EF-hand motifs within its structure. Apart from EF-hand-containing proteins, calcium-binding activity is observed in numerous other molecules, for instance, annexins [50], calreticulin [51] or calsequestrin [52], regucalcin [53], proprotein convertase subtilisin kexins (PCSKs) [54], or other proteins, which contain the C2-domain responsible for Ca^2+^ binding [55].

Modulation of calcium signalling occurs not only via cellular and membrane-bound molecules but also through extracellular calcium-binding proteins (ECBPs). This group of molecules plays a vital role in both extracellular calcium buffering and calcium sensing, providing a toolkit for the translation of calcium signals into a proper response. Similar to CBPs containing the EF-hand domain, many ECBPs also possess identifiable EF-hand motifs within their structure [56,57]. In addition, other high-affinity Ca^2+^-binding structures are observed, including EGF-like domains [58], γ-carboxyl glutamic acid-rich domains [59], cadherin domains [60,61], C-type lectin-like domains [62], and Ca^2+^-binding pockets of family C GPCRs [63,64]. Taken together, ECBPs create a highly heterogeneous family of Ca^2+^-binding proteins involved in molecular events such as the formation of binding sites for extracellular ligands, stabilisation of protein complexes or their anchoring to the cell membrane, receptor conformational changes, Ca^2+^-induced signal modulation, activation of downstream proteins, and finally signal transmission through a variety of transducers. ECBPs shape extracellular Ca^2+^ levels in the interstitial fluid of various organs, resulting in dynamic Ca^2+^ fluctuations. The shape and amplitude of observed Ca^2+^ variability appear to be a peculiar auto/paracrine mechanism of cell–cell communication essential for maintaining calcium homeostasis [65].

This review discusses the role of molecular components involved in the regulation of calcium homeostasis in the human uterus, with particular focus on their contribution to endometrial cancer.

## 3. Deregulation of Calcium Influx

### 3.1. Voltage-Gated Calcium Channels (VGCCs)

**Figure 2:**
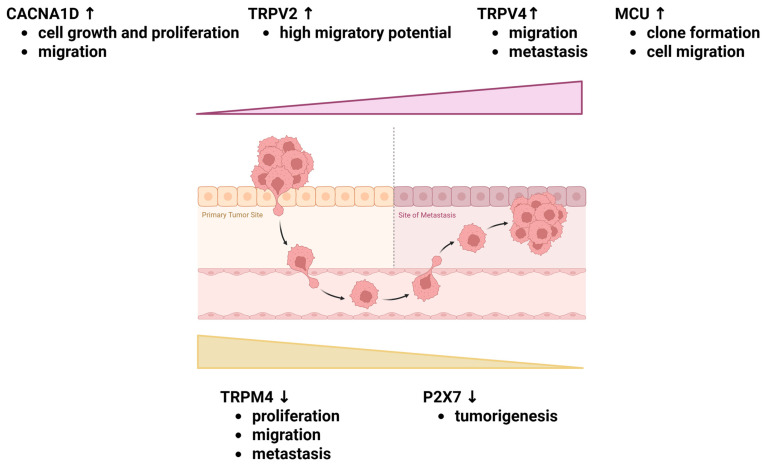
Dysregulated Ca^2+^ toolkit components associated with endometrial cancer progression. Upregulation of CACNA1D, TRPV2, TRPV4, and MCU promotes enhanced cell proliferation, migration, clone formation, and metastatic potential. In contrast, downregulation of TRPM4 and P2X7 reduces their tumour-suppressive roles, facilitating proliferation, migration, metastasis, and tumorigenesis. Collectively, these alterations in calcium signalling channels and transporters support tumour progression.

The voltage-gated calcium channels (Cav family) are ubiquitously expressed in human excitable cells; nonetheless, their role is also evident in non-excitable cells [34]. Based on their functional features, VGCCs are subdivided into low-voltage-activated (T-type or Cav3) and high-voltage-activated (L-type or Cav1, N-, P/Q-, and R-type or Cav2) types. Another classification based on structural similarities of channel-forming α1-subunit classifies VGCCs into L-(Cav1.1, Cav1.2, Cav1.3, and Cav1.4), P/Q-(Cav2.1), N-(Cav2.2), and R-(Cav2.3) channels, which form heteromultimers (along with auxiliary β-, α2δ, and γ-subunits) and T-type (Cav3.1, Cav3.2, and Cav3.3) channels, which are α1-subunit monomers [66]. The α1-subunit of VGCCs is encoded by the *CACNA1D* gene. As demonstrated, the expression of CACNA1D is under the control of oestrogens, as 17β-oestradiol treatment alleviates CACNA1D repression, at both mRNA and protein levels, after nifedipine blockade in HEC-1A cells [67]. Abrogation of CACNA1D in Ishikawa cells resulted in a significant decrease in intracellular Ca^2+^ concentration. Interestingly, immunohistochemical assessment demonstrated that CACNA1D expression is increased in atypical hyperplasia and cancerous tissues compared to benign endometrial lesions. Similar to studies in the HEC-1A cell line, Ishikawa cells treated with 17β-oestradiol demonstrated oestrogen-dependent regulation of CACNA1D expression in a dose- and time-dependent manner. However, siRNA experiment confirmed that the GPER is responsible for oestrogen-induced CACNA1D overexpression, rather than the canonical oestrogen receptors ERα and ERβ. Moreover, knockdown of CACNA1D resulted in a diminished calcium influx, which, in effect, inhibited cancer cell growth, proliferation, and migration [68] (Table 1, Figure 2).

The analysis of calcium-related genes based on the Cancer Genome Atlas (TCGA) dataset identified a four-gene signature of differentially expressed genes (DEGs) in endometrial cancer. This signature comprises *CACNA1D*, *SLC8A1*, *TRPM4* and *CCL2* genes. Elevated expression of *CACNA2D1* and *SLC8A1* genes was linked to EC progression, as reflected by cancer stage and grade, and correlated with poorer prognosis [69] (Table 1, Figure 2).

### 3.2. Transient Receptor Potential Channels (TRPs)

TRPs belong to the superfamily of over 30 ion channels, which are classified into six subfamilies based on sequence homology, i.e., TRPC (canonical), TRPV (vanilloid), TRPM (melastatin), TRPA (ankyrin), TRPP (polycystin) and TRPML (mucolipin). Activation of TRPs occurs in response to various signals, including temperature (heat and cold), as well as chemical and mechanical stimuli [88,89]. TRPs expression and function can be regulated/modulated by various hormones and growth factors present in the endometrial microenvironment. They are regarded as essential sensors that translate extracellular stimuli into cellular responses [90]. This phenomenon is supported by the fact that TRPs display distinct mRNA expression patterns in endometrial epithelial versus stromal cells [70,91]. High mRNA expression of TRPV2, TRPC1/4, and TRPC6 is characteristic of endometrial stromal cells, whereas in endometrial epithelial cells, the main TRP with pronounced expression is TRPV4, TRPV6, and TRPM6 [70,92]. The changes in TRPs expression have also been observed across different phases of the menstrual cycle [70,92]. Taken together, alterations in this strict balance in TRPs expression patterns may potentially contribute to cancerous transformation and progression of the human endometrium (Table 1).

A different pattern of TRPs expression during the menstrual cycle suggests sex-hormone involvement in the regulation/modulation of TRPs expression in the human endometrium. Functional studies have shown that TRPV1-mediated response to cannabinoids results in the reduction in cell viability via the activation of apoptosis in Ishikawa and Hec50co cell lines. Interestingly, induction of apoptosis was triggered by both endocannabinoids and cannabidiol, whereas Δ9-tetrahydrocannabinol had no impact on endometrial cancer cells. Stimulation of the apoptotic pathway was associated with TRPV1-mediated calcium influx. This pro-apoptotic activity was reversed upon treatment with the TRPV1 antagonist—5′-iodoresiniferatoxin (5-iRTX) [93].

On the other hand, immunohistochemical staining of TRPV2 has revealed a significant increase in type II endometrial cancer, which displays non-endometrioid histology. The observed upregulation has been associated with shorter progression-free survival in patients. In vitro, the upregulation of TRPV2 in Ishikawa cells resulted in higher migratory potential and a pronounced susceptibility to cisplatin-based treatment [73] (Table 1, Figure 2).

Further investigation revealed the TRPV4 channel as a significant player in the etiopathogenesis of endometrial cancer. The expression of TRPV4 was higher in cancerous tissue compared to normal endometrial epithelium [25] (Table 1). Functional studies have elucidated that TRPV4 contributes to endometrial tumorigenesis by regulating migration and metastasis (Figure 2). In vitro overexpression/depletion experiments supported this hypothesis. TRPV4 silencing reduced the migratory potential of Ishikawa cells, while exogenous TRPV4 expression enhanced cell motility, as demonstrated by a gap closure assay [25]. TRPV4-mediated calcium influx modulates endometrial cancer cell migration, both in vitro and in vivo, via the activation of the RhoA/ROCK1 pathway, which in turn orchestrates cytoskeletal remodelling [25]. The rapid regulation of cytoskeletal dynamics in HEC-1A and HEC-1B cells can be attributed to ERα signalling following E2 stimulation. Nonetheless, the data regarding E2-induced activation of TRPV4 expression, along with the subsequent cascade of calcium signalling, is limited [94]. On the other hand, an estrogenic stimulus activates the expression of TRPV6 channels in the human primary endometrial cells and the Ishikawa cell line. In the context of the menstrual cycle, the highest level of TRPV6, noted up to 1.5-fold, was observed during the secretory phase compared to the other phases [10] (Table 1).

The bioinformatics analysis of datasets from TCGA and Gene Expression Omnibus led to the development of a novel prognostic signature comprising 7 DEGs (differentially expressed genes) associated with endometrial cancer. *TRPM4* was identified as a constituent of the prognostic gene signature. The presence of the identified signature in patients with diagnosed endometrial cancer was associated with a favourable prognosis, as estimated by 1-, 3-, 5-, and 10-year overall survival rates [72]. The observed relationship between TRPM4 expression and a better prognosis was supported by the analysis of clinical characteristics of cancer samples. The lower levels of TRPM4 reflect an advanced clinical stage and tumour grade, which correlates with the presence of lymph node metastasis, myometrial invasion, as well as worse recurrence-free and overall survival (Figure 2). A similar observation was reported in another bioinformatic analysis, in which the *TRPM4* gene was included in a four-gene DEG signature that also included *CACNA1D*, *SLC8A1*, and *CCL2* genes [69] (Table 1).

Furthermore, in vitro experiments indicated that TRPM4 silencing enhanced proliferation and migration of AN3CA cells, accompanied by reduced p53 expression and a shift toward an EMT phenotype via hyperactivation of the PI3K/AKT/mTOR pathway [95]. Unlike TRPV4, TRPM4 expression is directly modulated by E2, as treatment with ER antagonists reversed its expression [95].

### 3.3. Ca^2+^ Release-Activated Ca^2+^ Channels

Ca^2+^ release-activated Ca^2+^ channels (CRAC) are the prototypical store-operated calcium channels (SOCs) involved in the mechanism of store-operated calcium entry (SOCE) [36]. SOCE induction occurs in response to Ca^2+^ depletion in the endoplasmic/sarcoplasmic reticulum (ER/SR) [96]. Calcium deficiency in ER/SR is sensed by the STIM (Stromal Interacting Molecule) sensor, whereas Ca^2+^ permeable pore formation in the plasma membrane involves the Orai protein. The functional STIM/Orai complex is called the CRAC. To date, three Orai isoforms (Orai1, Orai2, and Orai3) and two STIM proteins (STIM1 and STIM2) have been identified in mammalian cells [97]. The structurally and functionally characterised components of CRAC are the STIM1 and Orai1 molecules. Traversing the endoplasmic membrane, STIM1 senses reduced ER-luminal Ca^2+^ concentration via its N-terminal domain, where the EF-hand motif interacts with Ca^2+^ through electrostatic interactions. Upon Ca^2+^ depletion, STIM1 redistributes in the ER membrane, forming aggregates known as “puncta” that facilitate interaction with the cytoplasmic regions of Orai1 channel located in the plasma membrane [36]. As a result, the activated CRAC refills depleted endoplasmic Ca^2+^ stores by Ca^2+^ influx from the extracellular space [98].

Information on the significance of CRACsn uterine pathophysiology, particularly in carcinogenesis, remains limited. When comparing the spatio-temporal expression of Orai1 in human endometrium, the secretory phase is characterised by an elevated Orai1 expression (Table 1). Furthermore, it remains under the control of transforming growth factor TGFβ1, as the stimulation of Ishikawa cells with TGFβ1 leads to Orai1 upregulation, accompanied by a simultaneous increase in SOCE. A similar effect was observed when Ishikawa cells were transfected with constitutively active serum and glucocorticoid-inducible kinase-1 (SGK1). Taken together, the SGK1-sensitive regulation of Orai1 may be involved in the TGFβ1-mediated post-menstrual endometrial regeneration [74]. On the other hand, the LEFTY2, which also belongs to the TGFβ family, inhibited the endometrial receptivity by downregulation of Orai1, which in turn leads to the abrogation of SOCE [99].

Embryo implantation is a complex process orchestrated by ovarian-induced hormones, particularly progesterone, which facilitates endometrial receptivity. It has been demonstrated that modulation of cytosolic Ca^2+^ dynamics is progesterone-dependent, as progesterone upregulates the expression of the *ORAI1* and *STIM2* genes [100]. Knockdown of *STIM1* and *ORAI1*-*ORAI3* mRNA significantly impairs the rate of ER Ca^2+^ refilling, a phenomenon that overlaps with the effects observed upon TRPC1 silencing [101]. In pregnant women, the Orai2 encoding gene was the most abundantly expressed isoform in the human myometrium (Table 1). However, Orai1 appears to be engaged in calcium signalling regardless of labour onset, as it has been demonstrated that Orai1 regulation depends on the labour-associated cytokine IL-1β in primary human uterine smooth muscle cells [75]. An ex vivo immunohistochemical study of Orai isoforms in myometrium during pregnancy showed higher Orai1 expression in non-pregnant myometrium when compared to term pregnant myometrium [76] (Table 1).

### 3.4. Calcium/Cation Antiporters

Calcium/cation antiporters (CaCA) constitute a large, evolutionarily conserved superfamily comprising both potassium-independent and potassium-dependent transporters. Phylogenetic analysis divides the CaCA superfamily into five clades, three of which are expressed in mammalian cells, i.e., Na^+^/Ca^2+^ exchangers (NCX), K^+^-dependent Na^+^/Ca^2+^ exchangers (NCKX), and cation-Ca^2+^ exchangers (CCX) [102].

Na^+^/Ca^2+^ exchangers (NCX) are ubiquitous membrane-located antiporters expressed in different cell types. In mammalian cells, three NCX isoforms (NCX1, NCX2, and NCX3) have been identified, and they are distributed in both the plasma membrane and mitochondria. Each isoform is encoded by a separate gene (*SLC8A1-3*), which generates at least 17 alternative transcripts expressed in a tissue-specific manner [103]. NCX mediates the exchange of one Ca^2+^ for three Na+ ions, operating in either forward (Ca^2+^ efflux) or reverse (Ca^2+^ influx) mode, with the latter often linked to pathological conditions [104]. The activity of NCX depends on the net electrochemical gradients of Na^+^ and Ca^2+^, and its transport direction is determined by the membrane potential [105]. In turn, the activity of NCKX is K^+^-dependent, with a stoichiometry of four Na^+^ in exchange for one Ca^2+^ and one K^+^ ion. NCKXs are encoded by five genes (*SLC24A1-5*), and their protein products display organ and tissue specificity [106]. A distinct case within the Na^+^/Ca^2+^ exchanger family is the sixth member, encoded by the *SLC8B1* gene, which was initially referred to in the literature as NCKX6. However, since it is not K^+^-dependent, its current acronym is NCLX (Na^+^/Ca^2+^/Li^+^ exchanger) [107,108]. Based on recent findings, NCLX plays a critical role in releasing Ca^2+^ from mitochondria. Its activity is controlled by mitochondrial pH, calcium concentration, and membrane potential, and is also dependent on the kinase-mediated phosphorylation [109]. Although its exact stoichiometry and mechanism of action remain under debate, it is assumed that NCLX extrudes one Ca^2+^ in exchange for the uptake of three Na^+^ into the mitochondria [108].

Among the Na^+^/Ca^2+^ exchangers, NCX1 and NCKX3 are considered the most relevant for human uterine physiology [77]. NCKX3 has been shown to modulate endometrial receptivity, as its expression level increases 1.5–2.5-fold at both transcriptional and translational levels during the early- and mid-proliferative phases and the early- secretory phase of the menstrual cycle. Notably, such fluctuations were not observed in the case of NCX1 [77] (Table 1). The differential expression pattern of NCKX3 is related to the 17-β oestradiol (E2) stimulation since the treatment with ER-specific antagonists inhibited NCKX3 expression in the ER-positive cell line—Ishikawa, and was not evident in ER-negative cells—RL95 [77]. NCX1 and NCKX3 mRNA and protein were upregulated in both foetal and maternal sections of the placenta in preeclamptic tissue, in contrast to the normotensive placenta; however, this was only observed in cases of preterm labour. Tissue sections obtained from women during term labour were characterised by significant mRNA and protein downregulation of NCX1 and NCKX3. On the other hand, in vitro studies of NCX1 and NCKX3 expression in hypoxic conditions using isolated human placental cells during the first trimester revealed the elevation of calcium exchangers both at the levels of transcript and protein [78] (Table 1). To date, there is a lack of data regarding the involvement of CaCAs in the carcinogenesis of the human endometrium.

### 3.5. Purinergic Receptors

Purinergic receptors are widely expressed in mammalian cells and function as sensors of extracellular nucleotides. The purinergic signalling system operates through two classes of receptors: P1 (adenosine receptors) and P2 (nucleotide receptors) [110]. The first one comprises A1, A2A, A2B, and A3 receptors, while the second is divided into two types: P2X and P2Y. P2X and P2Y receptors act as ligand-gated ion channels, exhibiting distinct ion selectivity and unique signalling properties [111].

In mammals, seven P2X receptor subtypes have been identified (P2X1–P2X7) that form homo- or heterotrimers functioning as ligand-gated ion channels responsive to ATP. P2X subunits bind ligands via a domain located between two membrane-spanning regions (TM1 and TM2), with intracellular N- and C-termini. Upon ligand binding, P2X receptors induce an influx of Ca^2+^ and Na^+^ with simultaneous efflux of K^+^ [112,113].

P2X receptors have been shown to play diverse roles in the pathophysiology of human disease [111]. In the context of endometrial cancer, only the P2X7 receptors play a distinct role in the tumorigenesis of the uterine lining. In vivo studies demonstrated that P2X7 protein and mRNA levels were significantly reduced in complex hyperplasia with atypia or endometrial adenocarcinoma compared to normal endometrium, simple hyperplasia, or complex hyperplasia tissues [114]. Similar observations reported for the pre-cancerous (complex hyperplasia with atypia) and cancerous lesions suggest that decreased P2X7 expression may precede tumorigenesis of the uterus (Table 1, Figure 2). This decrease was not associated with reduced transcription of the *P2X7* gene, but rather resulted from enhanced degradation of its transcript [114]. Further information concerning the potential role of purinergic receptors in the development and progression of uterine pathologies in humans is limited to studies carried out using murine models. As the molecular biology of the uterus is closely linked to its specific structure and function, and the physiology of the rodent uterus differs significantly from that of the human uterus, this review does not discuss findings concerning murine models [79].

## 4. Deregulation of Calcium Extrusion to the Extracellular Space or Sequestration into ER/SR—Ca^2+^-ATPases

The P-type ATPases belong to a large superfamily of proteins divided into five groups (P1 to P5). P-type ATPases are ubiquitously expressed proteins associated with the plasma membrane, sarco/endoplasmic reticulum, and the Golgi apparatus. They exhibit broad substrate specificity, ranging from H^+^ to phospholipids [115], and some of them are specifically dedicated to the transport of Ca^2+^. Using energy stored in ATP, P-type ATPases transport ions across the lipid bilayer against their electrochemical gradient. Active shuttling of ions occurs through a series of different conformational states, each exhibiting distinct affinities for the transported ions. Transitions between high- and low-affinity states are triggered by phosphorylation and dephosphorylation of conserved aspartate residues. Due to conformational swinging, P-type ATPases are also called E1-E2 ATPases, a term coined after the intermediate E1-E2 topological state [116].

Each of the five subfamilies of P-type ATPases is further subdivided into subgroups (A, B, C, D, etc.). Among P-type ATPases, calcium homeostasis is primarily mediated by type P2 Ca^2+^-ATPases, both A and B. In mammalian cells, type P2A includes sarco/endoplasmic reticulum Ca^2+^ pumps (SERCA) and the secretory pathway Ca^2+^-ATPase (SPCA) located in the membranes of the Golgi apparatus. Type P2B, in turn, is represented by the plasma membrane Ca^2+^-ATPases (PMCA) [117]. The stoichiometry of calcium shuttling across membranes differs between these two subtypes. For SERCA, the hydrolysis of one ATP molecule is associated with the transport of two Ca^2+^ ions into the lumen of the ER, coupled with the countertransport of H^+^ to the cytosol. In contrast, PMCA extrudes one Ca^2+^ ion against an app. 10,000-fold Ca^2+^ gradient per one ATP molecule hydrolysed. PMCA pumps also demonstrate a unique regulatory property as they are activated by calmodulin (CaM), which binds to their C-terminal region, acting as a pump autoinhibitory domain [116].

Sarco/endoplasmic reticulum Ca^2+^ ATPases (SERCA) are predominantly expressed in the muscle ER membrane, reaching up to 50% of all expressed proteins. In human cells, SERCAs sequester cytosolic Ca^2+^ into the ER, which serves as the primary and easily accessible reservoir for intracellular calcium [118,119]. Three genes (*ATP2A1-3*) encoding SERCA pumps have been identified, and their expression is highly tissue-specific. Both isoforms of SERCA1, i.e., SERCA1a and SERCA1b, are characteristic of skeletal muscles, with SERCA1a predominantly expressed in adults and SERCA1b representing the neonatal form. SERCA2 is more ubiquitously expressed and is found in various tissues, depending on the variant, including muscle and neuronal cells (SERCA2a) and the heart (SERCA2c and SERCA2d). SERCA2b is the most abundant variant, serving as a housekeeping form [120,121]. The SERCA3 pumps, in turn, demonstrate high expression in hematopoietic-derived cells of the immune system, as well as in other cell types. The prominent role of SERCA3 in maintaining Ca^2+^ homeostasis is underscored by the identification of six alternatively spliced variants (SERCA3a-f) expressed in human cells. However, due to the numerous alternative variants of SERCA3, their particular roles in the development of human pathologies remain poorly understood [122].

The literature on the role of SERCAs in normal physiology or their contribution to the pathophysiology of the human uterus is scarce. The expression of SERCA2a and SERCA2b proteins has shown a significant increase in the myometrium of women in labour compared to those not in labour (Table 1). The myometrial contractility was diminished in the presence of SERCA2 inhibitor, i.e., cyclopiazonic acid (CPA) [80]. It is worth pointing out that the term nonlabour myometrium in the presence of a calcium-activated potassium channel inhibitor mimicked the active labour myometrium treated with CPA alone. Taken together, SERCA2 can be regarded as the primary component orchestrating calcium homeostasis, which is involved in maintaining myometrial contractile activity during labour [80]. Further studies suggest that the upregulation of SERCA2b observed during labour can be driven by interleukin 1β (IL-1β). Stimulation of human myometrial smooth muscle cells (HMSM) with IL-1β enhanced cell excitability, as demonstrated by increased basal calcium influx and the initiation of spontaneous calcium transients. In this study, IL-1β-mediated calcium mobilisation appears to be essential in preparing the pregnant uterus for labour [123]. Moreover, Ackerman et al. (2021), evaluating contraction-associated transcriptomic signatures in human labouring and non-labouring uteri, identified ATP2A2 (encoding SERCA2) and ATP2B4 (encoding PMCA4) genes as crucial transcripts shaping the labour phenotype [116] (Table 1).

On the other hand, SERCA2 is also engaged in the pathophysiology of endometriosis. It has been demonstrated that the development of endometriosis is accompanied by downregulation of long noncoding RNA maternally expressed gene 3 (LncRNA MEG3), particularly transcript MEG3-210. MEG3 is a tumour suppressor whose expression is significantly decreased in endometrial, cervical, and ovarian cancers [124,125]. This long noncoding RNA molecule interacts with various proteins, including galectin-1. Galectin-1 affects cell motility, signal transduction, and vascularisation and is overexpressed in both the ectopic and eutopic endometrium of women diagnosed with endometriosis [126]. Cellular consequences of MEG3-210 downregulation include the activation of p38 mitogen-activated protein kinase (p38 MAPK) and the inhibition of cAMP-dependent protein kinase A/sarcoplasmic reticulum Ca^2+^ ATPase 2 (PKA/SERCA2) signalling, which is mediated by galectin-1. Abrogation of PKA/SERCA2 signalling triggered by MEG3-210 downregulation alters the migration, invasion, and apoptosis of eutopic endometrial stromal cells (Eu-ESC). Moreover, SERCA2 downregulation alleviated the effects of MEG3-210 overexpression in Eu-ESCs. The molecule responsible for modulating p38 MAPK and PKA/SERCA2 signalling was galectin-1, as demonstrated using a mouse model of endometriosis. Nude mice were subcutaneously injected with human endometrial fragments, and those with MEG3-210 knockout developed significantly larger endometriotic lesions compared to controls. Interestingly, galectin-1 siRNA has been shown to reduce lesion size [127].

SPCAs, like SERCAs, are ATPases classified as type P2A. They are Golgi-localised pumps that play a crucial role in Ca^2+^ and Mn^2+^ transport, and SPCAs are also related to post-Golgi vesicles. To date, two SPCA isoforms, SPCA1 and SPCA2, have been identified in mammalian cells [128]. They are encoded by two distinct genes, *ATP2C1* and *ATP2C2*, and their polypeptide sequences share app. 60–65% similarity. Comparison of SPCA1 and SPCA2 aa sequences revealed that SPCA2 has a significantly longer N-terminus than SPCA1 [129]. Regarding their tissue distribution, SPCA1 is ubiquitously expressed, whereas SPCA2 shows more restricted expression, predominantly in the gastrointestinal and respiratory tracts, as well as in the prostate, thyroid, salivary glands, and mammary glands [130,131]. There are four transcript variants of SPCA1, which differ from each other in the C-terminal region, while no known transcript variations have been observed for SPCA2. Both SPCAs demonstrate similar kinetic properties, but SPCA2 exhibits distinctly lower Ca^2+^ affinity [132,133]. Despite the full length of the SPCA2 protein being app. 103 kDa, a shorter transcript encoding app. 20 kDa polypeptide chain was isolated from pancreatic acinar cells. The sequence of the short-length variants resembles the C-terminal region of the full-length SPCA2 protein [134].

The SPCAs’ specificity protects cells from Ca^2+^/Mn^2+^ overload by sequestering excess ions into the Golgi lumen. Furthermore, Mn^2+^ selectivity appears crucial for Mn^2+^ supply in the Golgi lumen, which is necessary for the proper functioning of glycosyltransferases and sulfotransferases. The structural elements responsible for metal ion transport during phosphoryl-transfer reactions are located within the N-terminus and transmembrane (TM) regions of SPCAs [132].

Based on the structural analysis of SPCA1, we can distinguish 10 transmembrane helices (TM1-TM10) assembled into three blocks: TM1-TM2, TM3-TM4, and TM5-TM10, which are related to conformational changes during the reaction cycle. On the cytosol-facing side, SPCAs possess three domains involved in ATPase activity: an A domain (connected with TM1-TM3), a P domain (linked to TM4 and TM5), and an N domain (connected to the P domain with two linkers, since it is located on the same polypeptide loop as the P domain). Interestingly, TM1, TM4 and TM6 regions are subdivided into sub-helices of C (cytosolic) and L (Golgi lumen) orientation [135]. SPCAs transport Ca^2+^ in a SERCA-like manner owing to a cytosolic headpiece interface consisting of the conserved TGE motif in the A domain, the phosphate-accepting Asp350 residue, the ATP- and FITC-binding region, and the DPPR loop located between the N and P domains [128].

There is no specific data concerning the role of SPCA in the uterus under physiological or pathological conditions. In the case of other tumours that remain under the control of oestrogen, such as mammary tumours, SPCA can cooperate with Orai1 to activate STIM1-independent calcium entry, which promotes breast tumorigenesis [136]. Additionally, studies in cancer cell models suggest that SPCA may initiate microcalcification in tumour lesions that correlate with cancer progression [137]. The basal-like breast cancer cells acquire a proliferative phenotype due to the upregulation of SPCA1. An increased growth rate results from elevated processing and trafficking of the insulin-like growth factor receptor (IGF-1R) [138]. Further studies on breast cancer cells revealed SPCA2 overexpression in the plasma membrane, where it directly activates Orai1 independently of STIM1. Activation of Orai1 triggers Ca^2+^ entry and induction of Ca^2+^-dependent proliferation [136]. The mouse xenograft model confirmed that SPCA2 silencing abrogated cell proliferation and anchorage-dependent breast tumour growth, accompanied by a concomitant reduction in resting Ca^2+^. In contrast, overexpression of SPCA2 produced the opposite effect. Surprisingly, the overexpression of SPCA2 mutants lacking ATPase activity also resulted in elevated resting Ca^2+^ and anchorage-dependent growth, as observed for the wild-type SPCA2. The tumorigenic activity of SPCA2, mediated by Orai1, was associated with the translocation of the NFAT transcription factor to the nucleus. Studies using chimaeras of SPCA2 have indicated that the SPCA2 N-terminus is crucial for interaction with Orai1 [136]. Conversely, it has been reported that SPCA2 couples Ca^2+^ influx via Orai1 to Ca^2+^ uptake into the Golgi/secretory pathway, which can be of high importance for shuttling Ca^2+^ into the secretory pathway of secreting cells, such as the mammary gland during lactation [139]. Taken together, SPCA transporters can be considered as a promising target for future anti-cancer strategies, exceeding beyond breast cancer treatment alone.

The last members of the P-type ATPase family are the plasma membrane Ca^2+^ ATPases (PMCA pumps), which play a crucial role in maintaining intracellular calcium homeostasis. Their primary role is to remove excess calcium ions from the cytosol into the extracellular matrix, thereby maintaining physiologically low levels of calcium ions in the cytoplasm [117]. In mammalian cells, four isoforms of PMCA pumps were identified, i.e., PMCA1, PMCA2, PMCA3, and PMCA4, encoded by four separate genes (*ATP2B1-4*) located at distinct chromosomes: 12q21.3, 3p25.3, Xq28 and 1q32.1, respectively [140,141]. *ATP2B1-4* genes can generate more than 30 splicing variants through alternative splicing, although only 20 are expressed in a cell- and tissue-specific manner. The diversity of expressed PMCA variants arises from tight regulation at transcriptional, splicing, translational, and protein levels [141,142,143].

PMCAs belong to the P-type ATPases, as evidenced by their structural and functional similarity to other representatives of this group. PMCAs are structurally similar to the closest relatives of P-type ATPases, i.e., sarco/endoplasmic reticulum type Ca^2+^ pumps (SERCAs, *ATP2A1-3* genes) with which they share an overall 30% sequence homology [144,145]. The structure of PMCA features a relatively large unstructured C-terminal region (30–130 residues depending on the isoform and variant) and four major domains: (1) the M-domain consisting of 10 trans-membrane spanning helices that provide coordinating ligands for binding a single cytosolic Ca^2+^ ion to be transported; (2) the N-domain, which binds an ATP molecule; (3) the P-domain with highly conserved aspartate residue responsible for forming a high-energy acyl-phosphate intermediate with phosphate group of hydrolysed ATP; (4) and the A-domain coordinating the M-, N-, P-domains during enzymatic reaction [115,141]. Hydrolysis of one ATP molecule provides sufficient energy to translocate one Ca^2+^ ion across the plasma membrane, with simultaneous coupled transport of H^+^ with a 1:2 ratio [146].

PMCA1 and PMCA4, due to their ubiquitous expression, are referred to as the “housekeeping isoforms”. In contrast, PMCA2 and PMCA3 are predominantly expressed in excitable cells. PMCA1 and PMCA4 display low basal activity, whereas PMCA2 and PMCA3 are characterised by high basal activity. Two alternative splicing sites, designated as site A and site C, can be identified within the genes encoding PMCAs [144]. Site A is located upstream of the phospholipid-binding site, while site C colocalises with the calmodulin-binding domain near the carboxy-terminal tail of the mature PMCA.

During the menstrual cycle, the *ATP2B1* gene is significantly upregulated only in the proliferative phase (early-, mid, and late phases), with its protein levels peaking in the early- and mid-proliferative phases (Table 1). The observed increase was accompanied by the upregulation of mRNA and protein expression of TRPV6, suggesting a prominent role of PMCA1 and TRPV6 in human reproduction. The PMCA1 expression is under the control of oestrogens, as evidenced by the significant increase in ATP2B1 mRNA following E2 treatment of the Ishikawa cell line. The aforementioned increase was abolished in the presence of the selective oestrogen receptor downregulator—fulvestrant [10]. During labour, PMCA1 expression is also upregulated in the human myometrium when compared to the myometrium of non-pregnant or non-labouring women (Table 1). However, PMCA overexpression is accompanied by an increase in SERCA2a and SERCA2b [80]. To date, no studies have reported on the role and function of PMCA1 in the cancerous transformation of the human uterus.

Transcriptomic expression of PMCA4 was found to correlate with the labour status of the human myometrium (quiescent vs. non-quiescent) only when analysed in relation to SERCA2 mRNA levels [81] (Table 1).

## 5. Calcium Release from Intracellular Reservoirs (Endoplasmic/Sarcoplasmic Reticulum)

ER and SR are the primary organelles that act as reservoirs of intracellular Ca^2+^ in both excitable and non-excitable cells. The first type of cells is predominantly characterised by the expression of RyRs (ryanodine receptors), whereas the latter relies on IP_3_Rs (inositol 1,4,5-trisphosphate receptors) to facilitate the release of Ca^2+^ from intracellular stores [147].

### 5.1. Ryanodine Receptors (RyRs)

RyRs are homotetrameric membrane channels consisting of approximately 5.000 amino acid residues. They were identified following the discovery that the alkaloid ryanodine, isolated from the South American plant Ryania speciosa, specifically targets RyR [148]. In mammals, RyRs are found in multiple cell types, including neurons, exocrine cells, epithelial cells, lymphocytes and others [149]. They are primarily involved in liberating Ca^2+^ from the SR, facilitating the excitation-contraction coupling essential for proper muscle contraction. To date, the literature has described three distinct RyR isoforms: RyR1, RyR2, and RyR3, which originate from the *RYR1*, *RYR2*, and *RYR3* genes, respectively [150,151,152,153,154]. All three RyRs isoforms are expressed in a tissue-specific manner. RyR1 is the predominant isoform in skeletal muscles, RyR2 expression is characteristic of cardiac cells, and RyR3, initially identified in the brain, is ubiquitously expressed. RyR1 and RyR2 are also present in neurons [155]. RyRs share app. 65% sequence similarity with a particular divergence attributed to D1, D2, and D3 regions, which are critical for calcium release and excitation-contraction coupling. In addition to the aforementioned regions of diversity, the large cytoplasmic domain (comprising four-fifths of the RyRs protein) contains sequences recognised by a wide array of regulators and modulators of its Ca^2+^ channel activity [149].

RyRs contribute to the modulation of uterine contractions in the human uterus, particularly within the myometrium [156,157]. Research has identified the expression of RyR isoforms in the human myometrium, including RyR1, which is primarily known for its role in skeletal muscle. Its expression remains relatively constant throughout pregnancy, suggesting a role in maintaining uterine contractility during labour [82]. The expression of RyR2, an isoform typically associated with cardiac muscle, is minimal in the non-pregnant myometrium but increases during pregnancy [84]. This upregulation may enhance the uterus ability to contract at term, indicating a potential role in preparing the uterus for labour. Conversely, RyR3 is expressed in both non-pregnant and pregnant myometrium and is the predominant isoform in these tissues. Its consistent expression suggests a fundamental role in uterine muscle function [82,84] (Table 1). The differential expression of these RyR isoforms during pregnancy suggests a finely tuned mechanism through which the uterus regulates calcium dynamics to control muscle contraction, which is essential for labour and delivery. Interestingly, the expression of RyR2 and RyR3 in myometrial cell cultures can be restored after the treatment with TGFβ, suggesting that TGFβ-induced RyR2 expression might be essential for the maturation of the human myometrium during pregnancy in preparation for labour [84]. Additionally, several studies have explored the impact of RyR mutations on uterine function. For instance, it has been demonstrated that specific mutations in the *RYR1* gene can influence uterine artery vasodilation and myometrial contraction, potentially leading to complications such as abnormal bleeding and altered foetal-placental growth [158].

In summary, ryanodine receptors are integral to regulating uterine contractions, with their expression and function adapting throughout pregnancy to facilitate successful labour. In human myometrial cells, RyRs also play a significant role in mediating oxytocin-induced intracellular calcium transients alongside the classical inositol trisphosphate (IP_3_)-dependent pathway. Wray et al. (2007) demonstrated that cyclic ADP-ribose (cADPR), a known RyR activator, induces calcium release from intracellular stores via RyRs, and that pharmacological inhibition of RyRs substantially attenuates oxytocin-evoked calcium signals [157]. Moreover, the pro-inflammatory cytokine TNF-α upregulates CD38 expression, thereby enhancing cADPR production and potentiating RyR-mediated calcium release. These findings suggest that RyRs, modulated by the cADPR pathway, play a role in regulating uterine contractility and may serve as a target for the development of new therapies to treat abnormal myometrial contractions during pregnancy [159].

When considering the role of RyRs in the pathophysiology of other structures of the human uterus, Worton et al. (2021) demonstrated that RyRs inhibition by ryanodine does not abolish the vasorelaxant response to kynurenine on vascular smooth muscle cells from omental arteries isolated from normotensive pregnant women [160]. Additionally, human endometrial stromal cells (ESCs) treated with dantrolene, an inhibitor of RyRs, exhibited increased expression of decidual marker genes, i.e., *PRL* (prolactin) and *IGFBP1* (IGF-binding protein 1). This observation suggests that fluctuations in cytosolic Ca^2+^, mediated by changes in intracellular cAMP levels, may regulate the differentiation of endometrial stromal epithelial cells [161]. Interestingly, RyR1 mRNA and protein expression were significantly upregulated in uterine fibroids when compared to myometrial tissues (Table 1). Concomitantly, increases in Ca^2+^ and cytochrome c levels were observed, likely reflecting elevated cell apoptosis, as evaluated by flow cytometry using annexin V and PI. Under hypoxic conditions, fibroid cells were much more susceptible to apoptosis than adjacent myometrial cells. The induction of apoptosis was effectively blocked using a RyR blocker, ruthenium red. Taken together, RyR-mediated calcium release can be regarded as a crucial stimulus of apoptosis in uterine fibroids [83].

### 5.2. Inositol 1,4,5-Trisphosphate Receptors (IP_3_Rs)

To date, three isoforms of IP_3_Rs have been identified, i.e., IP_3_R1, IP_3_R2, and IP_3_R3. All three isoforms are encoded by separate genes (*ITPR1–3*), and due to their different ligand affinities, they exhibit similar yet distinct functional properties, contributing to their roles in diverse cellular processes [162]. IP_3_Rs are tetrameric intracellular Ca^2+^ channels located in the membrane of the ER that release Ca^2+^ upon binding of IP_3_. IP_3_R assemblies may consist of the same or different subtypes of monomers. Structurally, IP_3_Rs isoforms share up to 70% homology, with even higher similarity, up to 90% identity, within the TM regions, whereas the regulatory cytoplasmic domains display considerable diversity [163]. Cryo-EM studies have revealed that the IP_3_R structure consists of three distinct domains connected by slender linkers, exhibiting a flower-like appearance and fourfold symmetry [163,164]. IP_3_R transmembrane regions are responsible for the pore formation, whereas their cytoplasmic domains contain binding sites for ligands and modulators. The channel’s activation involves IP_3_ binding, which primes the receptor to bind Ca^2+^, triggering channel opening. IP_3_Rs exhibit unique properties, such as multiple cavities, dynamic conformational changes, and the ability to retain function even when fragmented. IP_3_Rs’ activity, in cooperation with phospholipase C-dependent signalling, plays crucial roles in diverse cellular and tissue-level processes, including apoptosis, exocrine secretion, fertilisation, hormone secretion, immune response, metabolic regulation, and neuronal plasticity [163,164]. Studies on expression patterns have revealed that all three IP_3_R isoforms differ from each other in tissue distribution, and they display different affinity for IP3 (IP_3_R2 > IP_3_R1 > IP_3_R3). Although IP3Rs are located in the endomembrane, the signal initiation for Ca^2+^ release from the ER originates at the plasma membrane, where G-protein-coupled receptors, activated upon binding their agonists, trigger the activation of PLC. PLC then hydrolyses phosphatidylinositol 4,5-bisphosphate (PIP_2_), generating diacylglycerol (DAG) and the second messenger IP_3_, which in turn activates its dedicated receptors located in the ER-membrane [165].

Alterations in IP_3_Rs that disrupt Ca^2+^ homeostasis have been linked to various neurodegenerative and neuromuscular diseases, as well as cancer [38,166,167]. In the context of the human uterus, the available studies provide limited insights into the expression and functional roles of IP_3_Rs in uterine physiology and pathology. The earliest report on human myometrium has demonstrated that IP_3_Rs play a pivotal role in regulating calcium signalling, which is essential for uterine contractility [85,168]. The presence of high-affinity IP_3_ binding sites has been detected in human myometrial tissue irrespective of pregnancy status, suggesting that IP_3_Rs are constitutively expressed in the uterus (Table 1). Furthermore, stimulation with oxytocin leads to increased production of IP_3_, which then binds to its receptors on the endoplasmic reticulum, initiating the release of Ca^2+^ into the cytosol. This rise in intracellular calcium concentration subsequently triggers myometrial contraction. Thus, IP_3_Rs are central to the signal transduction cascades linking hormonal stimulation to uterine smooth muscle activation, highlighting their fundamental role in both physiological uterine function and potential pathological conditions related to abnormal contractility. Further studies have revealed that all three IP_3_R transcripts are present in human myometrial smooth muscle, although their levels vary among individuals [169]. Brighton et al. (2020) [170] demonstrated that retosiban, a small-molecule oxytocin receptor antagonist, attenuates oxytocin-induced Ca^2+^ release and muscle contraction by targeting the IP_3_R-mediated Ca^2+^ mobilisation pathway. This mechanism of action, characterised by a lack of coupling of the oxytocin receptor to Gαi with subsequent phosphorylation of ERK 1/2, and upregulation of COX2, and secretion of PGE2 and PGF2α, suggests that retosiban may offer a more effective approach for uterine relaxation or prevention of preterm labour than the currently used peptide-based compound atosiban. In contrast to retosiban, atosiban promotes inflammatory responses and stimulates the secretion of prostaglandins characteristic of parturition [170].

Studies on blastocyst implantation unravelled that IP_3_Rs are involved as downstream effectors of PAR2 (protease-activated receptor 2) in the depletion and refilling of intracellular stores in response to calcium signals released by the blastocyst [171]. Furthermore, altered intracellular Ca^2+^ concentration and modulation of ion channel gene expression are involved in the proliferation and differentiation of hEMSCs (human endometrial stromal cells), resembling cellular processes during decidualization. Combined progesterone and cyclic AMP stimulation of hEMSCs not only induces IP_3_R expression but also triggers IP_3_R-mediated Ca^2+^ release [100]. In human endometrium-derived stem cells exposed to oxidative stress (H_2_O_2_), rapid intracellular Ca^2+^ elevation is mediated by the PLC–IP_3_–IP_3_R pathway, triggering Ca^2+^ release from ER stores. Pharmacological blockade of IP_3_Rs with 2 APB (2-aminoethoxydiphenyl borate) significantly reduces the Ca^2+^ surge, implicating IP_3_R as the primary mediator of this response. Sustained Ca^2+^ elevations drive cellular senescence, whereas buffering Ca^2+^ with BAPTA AM shifts the outcome toward AMPK-dependent autophagy. Thus, IP_3_R functions as a pivotal switch between senescence and autophagy in stressed endometrial stem cells [172].

Similar to RyRs, increased IP_3_R1 expression, at both transcript and protein levels, has been observed in uterine fibroids compared to adjacent myometrial tissues. Furthermore, this upregulation appears to increase the susceptibility of fibroid cells to apoptosis under uterine artery occlusion by laparoscopy [83].

## 6. Mitochondrial Ca^2+^ Uptake

### Mitochondrial Ca^2+^ Uniporter Complex (MCU Complex)

Mitochondria possess a high capacity for shaping intracellular Ca^2+^ homeostasis, thereby actively modulating Ca^2+^ signalling. Key components regulating mitochondrial Ca^2+^ concentration include the mitochondrial calcium uniporter (MCU), the voltage-dependent anion channel (VDAC), and the mitochondria-associated ER membranes (MAMs) [173]. Among them, only MCU has been reported as a potential contributor to calcium homeostasis in pathologies originating from the human uterus [86,87]. The MCU is a Ca^2+^-specific uniporter that facilitates the uptake of Ca^2+^. It forms a tetrameric complex consisting of three different types of subunits: MCU (pore-forming subunit), MCUb (MCU dominant negative subunit beta), and EMRE (essential MCU regulator) [174,175,176,177]. The MCU is ubiquitously expressed 40 kDa protein localised in the inner mitochondrial membrane (IMM). Structurally, it possesses two transmembrane domains linked by a loop facing the intermembrane space. The loop is negatively charged due to the presence of acidic amino acid residues, and its sequence is highly conserved [176,177]. A similar structure is observed for MCUb, an alternative isoform of MCU that shares 50% sequence homology. The main structural difference influencing the MCUb conductivity is the presence of an amino acid substitution in the loop region (E256V) that neutralises its negative charge [178]. The MCU/MCUb ratio, which varies among different tissue types, appears to determine the mitochondrial Ca^2+^ uptake capacity, thereby enabling cells to adapt to their specific physiological functions [174]. The EMRE subunit plays a critical role in the function of the MCU complex. The EMRE is a single transmembrane 10 kDa protein located in the IMM. The absence of EMRE abolishes MCU complex activity. Functional studies revealed that EMRE connects the pore-forming complex with regulatory proteins, such as MICUs (mitochondrial calcium uptake) [175]. The MICU1-3 regulators are responsible for sensing Ca^2+^ levels. These proteins, located in the intermembrane space (IMS), potentiate the activity of the MCU holocomplex when extramitochondrial Ca^2+^ levels are elevated. MICUs modulate the gating properties of the MCU complex by promoting its open state [179,180].

The studies focusing on the role of MCU in human uterus physiology have reported its potential involvement in gestation, with altered expression possibly contributing to preterm births. Vishnyakova et al. compared the expression profiles of MCU holocomplex components in the placenta and myometrium, key elements of the foeto-maternal system, at full-term delivery and preterm birth. The evaluation of MCU, MCUb, EMRE (also known as SMDT1), and MICU1-2 expression, both at the transcriptomic and proteomic levels, revealed dynamic changes throughout gestation. MCU and MICU1 subunit expression demonstrated a gradual increase in the placenta during gestation (Table 1). The mitochondria isolated from placentas at preterm birth were characterised by a slower depolarisation ratio. In the myometrium during preterm birth, the relative expression levels of genes encoding MCU, MCUb, and EMRE were elevated compared to full-term pregnancy. Gestational dynamics revealed a gradual increase in MCU and MCUb protein levels, accompanied by a decline in MICU1 expression [86] (Table 1). Taken together, the diverse expression profiles of MCU complex components may reveal novel molecular targets for treatment strategies aimed at reducing the risk of premature birth.

Regarding the contribution of MCU to the development and progression of endometrial cancer, only one study has reported the role of MCU in this tumour type [87]. Mitochondrial calcium uniporter (MCU) is markedly upregulated in endometrial cancer tissue compared to normal endometrium, with elevated MCU levels strongly correlating with higher histological grade, deeper myometrial invasion, and lymph node metastasis (Table 1). Using Ishikawa and RL95-2 cell lines, it was demonstrated that MCU physically interacts with voltage-dependent anion channel 1 (VDAC1), forming a functional complex that enhances mitochondrial calcium uptake and mitochondrial activity. These alterations promote oncogenic features, including increased clone formation, enhanced cell migration, and stabilisation of β-catenin (Figure 2). Interestingly, silencing of MCU or VDAC1 via shRNA reduced the expression of NCLX and β-catenin, thereby inhibiting tumour cell proliferation and motility. These findings implicate MCU and its interaction with VDAC1 as key regulators of mitochondrial Ca^2+^ handling in endometrial carcinogenesis, positioning the MCU-VDAC1 axis as a promising therapeutic target [87].

## 7. Potential Targets for Novel Treatment Strategies of Endometrial Cancer

Recent research has highlighted the potential of targeting calcium homeostasis for the treatment of endometrial cancer. Intracellular calcium ions play a crucial role in cancer progression, with oestrogen-induced calcium increase affecting cell proliferation and migration [69]. High serum calcium levels correlate with poor clinical outcomes in endometrial cancer patients, and oestrogen-induced extracellular calcium influx regulates mitochondrial ROS and lysosomal activity [30]. Dysregulation of calcium handling proteins in cancer cells disrupts normal calcium flux, affecting key signalling pathways involved in cancer cell proliferation and invasion [181]. Calcium channel blockers have shown potential in regulating oestrogen-induced intracellular Ca^2+^ rises and cell behaviour, suggesting their possible repurposing for endometrial cancer treatment [69].

**Figure 3:**
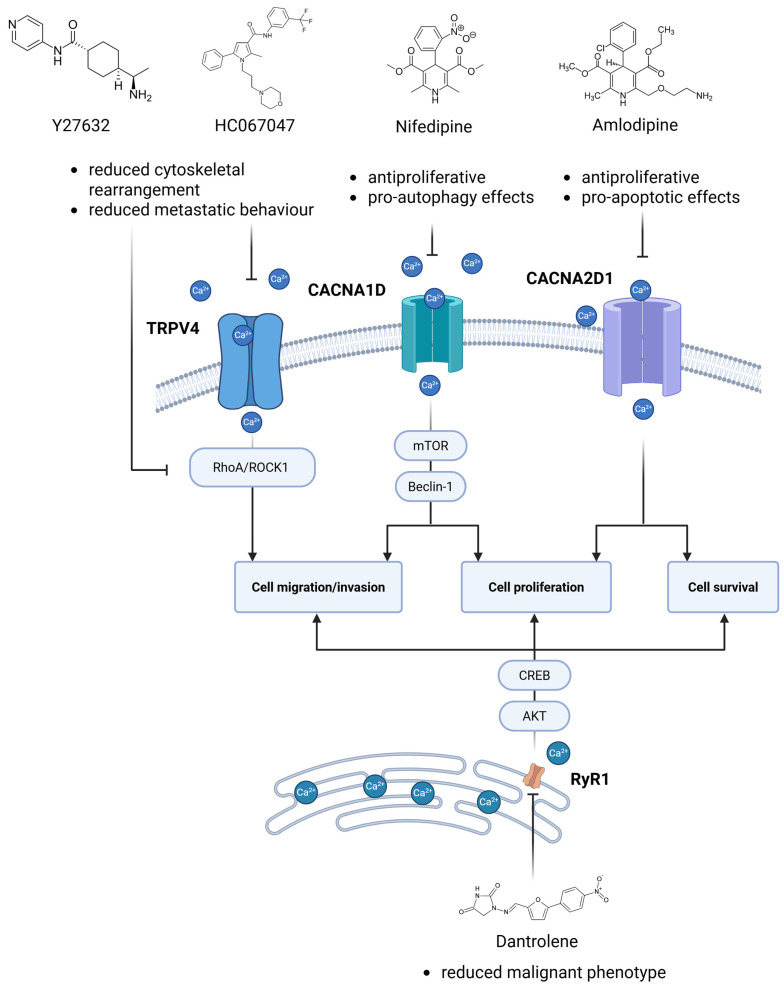
Potential strategies based on small-molecule inhibitors targeting Ca^2+^ homeostasis components in endometrial cancer. Selective inhibitors that modulate Ca^2+^ channels and transporters may have potential applications in developing novel strategies for endometrial cancer treatment.

The potential of targeting calcium signalling pathways as a therapeutic strategy in endometrial cancer is also highlighted by recent preclinical studies. Although no clinical trials have directly focused on calcium channels or transporters in EC to date, several calcium-regulating proteins have emerged as promising targets. Notably, the overexpression of CACNA2D1, an α2δ subunit of voltage-gated calcium channels, has been linked to a poor prognosis, and its pharmacological inhibition using amlodipine, a clinically approved calcium channel blocker targeting α_2_δ_1_, has been shown to reduce proliferation and induce apoptosis in EC models (Figure 3). In vitro knockdown of CACNA2D1 via siRNA significantly inhibited the proliferation and migration of EC cell lines, and pharmacological blockade with amlodipine, reproduced these inhibitory effects. Importantly, in vivo treatment with amlodipine led to significant tumour growth suppression in xenograft models [69]. These findings support the translational potential of amlodipine repurposing as a novel adjunct therapy in EC by targeting dysregulated Ca^2+^ influx mediated by CACNA2D1. Similarly, CACNA1D, which is responsive to 17β-oestradiol, promotes proliferation and migration. In contrast, antagonists like nifedipine demonstrate antiproliferative and pro-autophagic effects. Treatment with nifedipine (10 µM) markedly increased the formation of autophagic vacuoles (monitored by GFP LC3 and MDC staining) and elevated Beclin 1 levels, implicating activation of the Beclin 1/mTOR axis. The autophagy induced by nifedipine was inversely correlated with CACNA1D expression, suggesting that calcium influx through L-type channels might regulate downstream signalling involved in autophagy (Figure 3). Although IP_3_R was not directly investigated, the results suggest that blocking plasma membrane Ca^2+^ entry may reduce IP_3_-mediated Ca^2+^ release from the ER, thereby shifting calcium-dependent signalling toward autophagy rather than proliferation or apoptosis. These findings support the potential therapeutic value of calcium channel antagonists in EC and underscore the need to explore IP_3_R-related pathways in future studies [182].

Recent findings suggest that cannabinoids, including anandamide (AEA) and cannabidiol (CBD), may hold therapeutic potential in the treatment of endometrial cancer [93]. In vitro studies using Ishikawa and Hec50co cell lines demonstrated that AEA and CBD significantly reduced cancer cell viability. However, only Ishikawa cells presented reduced cell viability through mechanisms involving apoptosis, characterised by caspase-3/7 activation, PARP cleavage, and increased reactive oxygen species production. Notably, these effects were mediated by TRPV1 receptor activation and calcium influx, indicating that cannabinoid-induced modulation of calcium signalling may be adopted as a novel anti-tumour strategy. The involvement of TRPV1 in mediating these responses highlights a promising pharmacological target for the development of cannabinoid-based therapies against oestrogen-dependent endometrial cancer [93]. Further studies, concerning the potential utility of CBD in the treatment of endometrial cancer, have shown reduced cell viability in multiple endometrial cancer cell lines in a dose-dependent manner, promoting apoptosis in type I endometrioid lines and autophagy in mixed-type models. Notably, CBD enhanced the cytotoxic effects of chemotherapeutic agents, particularly in the presence of TRPV2 overexpression. These findings position CBD as a promising adjuvant therapeutic agent for endometrial cancer, especially in tumours exhibiting elevated TRPV2 [73].

TRPV4, a calcium-permeable channel, is implicated in metastatic behaviour, and its inhibition reduces invasiveness in vitro. Recent research has shown that TRPV4 plays a crucial role in promoting metastasis in endometrial cancer by regulating calcium influx and cytoskeletal dynamics. Elevated levels of Ca^2+^ in serum correlate with high TRPV4 expression in endometrial cancer patients, and both are associated with the advanced stage of disease and poor prognosis. Mechanistic studies in HEC 1A and Ishikawa cell lines demonstrated that TRPV4-mediated calcium entry activates the RhoA/ROCK1 signalling cascade, leading to phosphorylation of LIMK and cofilin, reorganisation of F-actin stress fibres and paxillin, and enhanced cell migration/invasion. Pharmacological inhibition using a TRPV4 antagonist (HC067047), calcium chelator (BAPTA-AM), or ROCK inhibitor (Y27632) effectively blocked cytoskeletal changes and reduced metastatic behaviour (Figure 3). These results position TRPV4 and its downstream calcium-dependent pathways as promising therapeutic targets for inhibiting metastatic progression in endometrial cancer [25].

Targeting RyR1 represents another potential therapeutic strategy. In uterine serous carcinoma (USC), the most aggressive subtype of uterine cancer, elevated RyR1 expression has been shown to correlate with advanced disease stage and poor prognosis. Notably, treatment with dantrolene significantly reduced malignant phenotypes in USC cell lines ARK1, ARK2, and Hec50co. Dantrolene is a specific blocker of RyR1 and RyR3, approved by the FDA for the treatment of conditions such as malignant hyperthermia (MH) and central core disease (CCD). A similar effect was obtained using genetic knockdown of RyR1 via shRNA. Pharmacological or genetic inhibition of RyR1 resulted in diminished proliferation, migration, and colony-forming ability, both in vitro and in xenograft mouse models, accompanied by an increase in apoptosis. Mechanistically, dantrolene attenuated RyR1-mediated calcium release and downstream signalling pathways involving AKT, CREB, and PGC 1α, and disrupted mitochondrial reprogramming essential for tumour cell survival (Figure 3). However, these findings support dantrolene-based strategies only in RyR1-overexpressing endometrial malignancies [183].

Similarly, blockade of MCU, which is overexpressed in more advanced endometrial cancers, reduces tumour growth, clone formation, migration, and mitochondrial activity in vitro. Conversely, MCU overexpression enhances these malignant properties. Silencing of either MCU or VDAC1 resulted in reduced expression of MCU, VDAC1, NCLX, and β-catenin. Furthermore, VDAC1 knockdown attenuated the enhancing effect of MCU overexpression on these proteins. Collectively, this study demonstrates that MCU-mediated mitochondrial calcium uptake plays a pivotal role in endometrial tumorigenesis, and MCU interaction with VDAC1 can be regarded as a potential target for pharmacological interventions in endometrial cancer [87]. Nonetheless, the application of MCU inhibitors as a novel therapeutic approach in endometrial cancer has not yet been explored [184].

In type I (oestrogen-dependent) endometrial cancer, the calcium-oestrogen axis is particularly pronounced, reflecting higher expression of oestrogen receptors and calcium-regulatory genes, whereas type II tumours, typically oestrogen-independent, exhibit attenuated responsiveness to this signalling network [185]. Understanding this receptor–channel–signalling hierarchy provides a mechanistic rationale for targeting the oestrogen–calcium axis therapeutically. Combining oestrogen receptor antagonists, e.g., fulvestrant and tamoxifen, with calcium-modulating agents, such as voltage-gated channel blockers or SOCE inhibitors, may offer a synergistic strategy to counteract aberrant calcium signalling and reduce tumour growth potential [27,186]. Targeting the oestrogen-calcium axis shows potential as a therapeutic strategy for endometrial cancer, but evidence is currently limited to preclinical research. The available evidence suggests this approach holds promise. Research demonstrates that oestrogen can rapidly increase intracellular calcium through membrane-initiated effects, activating kinase cascades that may regulate cancer initiation, progression, and metastasis [29]. Studies indicate that inhibiting calcium ion channel proteins could serve as adjuvant therapy, with calcium channel blockers reportedly regulating both oestrogen-induced calcium increases and key cancer processes, including cell proliferation, apoptosis, and migration [28].

Based on current evidence that CACNA1D is upregulated in oestrogen receptor-positive endometrial cancers and that L-type channel blockers such as nifedipine can reduce proliferation and migration in vitro. Combined treatment including CACNA1D inhibitors and oestrogen receptor antagonists could yield synergistic anti-tumour effects [29,187]. This combination could simultaneously suppress oestrogen-induced upregulation of calcium channels and downstream proliferative signalling while blocking channel-mediated calcium influx. This strategy might be particularly effective in type I endometrial cancer, which is hormone-responsive, and could be explored in preclinical models using agents such as fulvestrant or pure oestrogen receptor antagonists in conjunction with Cav1.3 blockers. The described clinical case, including the report of combining amlodipine with letrozole, indicates that calcium channel blockade along with estrogen suppression is well tolerated and may be effective during maintenance after chemotherapy [188].

In addition to CACNA1D, CACNA2D1 has emerged as another promising auxiliary component of VGCCs in endometrial cancer since high CACNA2D1 expression is associated with higher grade, advanced stage, poorer survival, and enrichment of estrogen-response signaling. Knockdown of CACNA2D1 or its pharmacologic blockade using amlodipine reduces proliferation, induces cell cycle arrest, and suppresses tumour growth in in vivo models [69]. Given these findings, combining CACNA2D1 inhibitors with ER antagonists could provide additive or synergistic suppression of EC growth by blocking both the stimulus (i.e., the estrogen-ER axis) and the enabler that enhances calcium influx via VGCCs. This strategy warrants preclinical validation, especially to assess subtype specificity (type I vs. type II) and optimal dosing to minimize off-target effects.

Despite compelling pre-clinical evidence linking dysregulated calcium homeostasis to endometrial cancer progression, no drugs targeting calcium-regulating proteins have yet been translated into clinical practice. Several factors contribute to this gap, including broad expression and a lack of specificity. Calcium channels, pumps, and exchangers are widely expressed in multiple tissues, including the heart, neurons, and smooth muscle, where they play essential roles in maintaining physiological functions. Non-selective inhibition could therefore cause severe adverse effects such as arrhythmias or neurological dysfunction. The next issue covers the limited availability of selective inhibitors. Current pharmacological blockers, such as classical calcium channel blockers used in cardiology, lack specificity for particular subtypes of TRP channels, Orai/STIM complexes, or mitochondrial transporters (MCU) implicated in endometrial cancer. It may potentially hamper their oncological use. The third problem can be addressed to complex and redundant signalling networks. Calcium signalling is highly dynamic and redundant. Cancer cells may compensate for the inhibition of one pathway by upregulating alternative calcium entry routes, reducing the efficacy of targeted therapies. Although extensive in vitro and animal model data exist, all candidates have been studied at the pre-clinical stage. Barriers also include safety concerns, inadequate pharmacokinetics, and challenges in designing oncology-focused studies for this class of targets. Lastly, calcium signals in endometrial cancer may exert both pro-proliferative and pro-apoptotic influences, depending on the cellular context, tumour stage, and microenvironment. This duality complicates straightforward therapeutic targeting. Taken together, these challenges explain why no calcium-targeting drugs have yet been clinically implemented for endometrial cancer. Significantly, the absence of approved therapies does not diminish the biological relevance of the calcium toolkit. Instead, it highlights the need for more selective modulators, improved translational models, and combined therapeutic approaches, such as those with chemotherapy or immunotherapy, which may eventually enable clinical application.

## 8. Conclusions

As outlined in this review, deregulation of the cellular calcium machinery is a recurring feature in endometrial cancer. The observed alterations in calcium signalling are reported across various cellular compartments and involve a wide range of transporters, channels, and pumps localised in the plasma membrane, sarcoplasmic/endoplasmic reticulum (SR/ER), and mitochondrial membranes. Dysregulated expression or function of these components can disrupt the tightly controlled balance of calcium influx and efflux, leading to aberrant intracellular calcium dynamics that contribute to cancer proliferation, migration, survival, and resistance to therapy. Targeting these dysregulated components may enable selective modulation of calcium signalling pathways that are essential for tumour growth and resistance to apoptosis. This opens the possibility for developing more precise, mechanism-based treatment strategies for endometrial cancer, including small-molecule inhibitors, ion channel modulators, targets of the endocannabinoid system or gene-silencing approaches, aimed at restoring calcium homeostasis and inhibiting disease progression. An interesting direction in developing new therapies for oestrogen-dependent endometrial cancer involves using calcium channel blockers alongside anti-oestrogen drugs as adjuvant treatments.

Moreover, the limited literature data regarding the involvement of calcium homeostasis components, such as SERCA, SPCA, STIM, or TRPC, in the development and progression of endometrial cancer should not deter further research. Instead, it should indicate potential new directions for studies on therapeutic targets based on these hitherto unexplored proteins involved in maintaining proper intracellular Ca^2+^ levels in endometrial cancer.

## Figures and Tables

**Table 1:** Expression of Ca^2+^ homeostasis components in the human uterus.

Transporter/Channel/Pump	Tissue	Changes	Comments	Ref.
**VGCC**				
**CACNA1D**	atypical hyperplasia, cancerous tissue	increased	IHC; vs. benign endometrial lesions	[68]
**CACNA2D1**	endometrial cancer (TCGA-DEG)	increased	mRNA vs. normal endometrium	[69]
**TRP**				
**TRPC1/4**	endometrial stroma		mRNA	[70,71]
**TRPC6**	endometrial stroma		mRNA	[70,71]
**TRPM4**	endometrial cancer (TCGA-DEG)	decreased	lower level—poorer prognosis vs. normal endometrium	[69,72]
**TRPM6**	endometrial epithelium		mRNA	[70,71]
**TRPV2**	non-endometrioid cancer	increased	IHC; shorter progression-free survival vs. normal endometrium	[73]
**TRPV2**	endometrial stroma		mRNA	[70,71]
**TRPV4**	endometrial cancer	increased	IHC vs. normal endometrium	[25]
**TRPV4**	endometrial epithelium		mRNA	[70,71]
**TRPV6**	endometrial epithelium		mRNA	[70,71]
**TRPV6**	normal endometrium		secretory phase	[10]
**Orai/STIM**				
**Orai1**	normal endometrium	increased	IHC; secretory phase vs. other phases	[74]
**Orai2**	myometrium		mRNA	[75]
**Orai2**	myometrium	increased	non-pregnant vs. pregnant	[76]
**CaCA**				
**NCKX3**	normal endometrium	increased	mRNA; IHC; early-, mid-proliferative phases; early-secretory phase vs. other phases	[77]
**NCKX3**	fetal and maternal placenta	increased	mRNA; IHC; preeclamptic tissue preterm labour	[78]
**NCKX3**	fetal and maternal placenta	decreased	mRNA; IHC; term labour	[78]
**NCKX3**	isolated placental cells (1st trimester)	increased	mRNA; WB; 1st trimester; hypoxic conditions	
**NCX1**	fetal and maternal placenta	increased	mRNA; IHC; preeclamptic tissue preterm labour	[78]
**NCX1**	fetal and maternal placenta	decreased	mRNA; IHC; term labour vs. preterm birth	[78]
**NCX1**	isolated placental cells (1st trimester)	increased	mRNA; WB; hypoxic conditions vs. normoxic conditions	[78]
**Purinergic receptors**				
**P2X7**	complex hyperplasia with atypia, endometrial adenocarcinoma	decreased	mRNA; IHC; vs. normal endometrium, simple hyperplasia, complex hyperplasia	[79]
**P-type ATPases**				
**SERCA2(a/b)**	myometrium	increased	WB; labour vs. non-labour	[80]
**PMCA1**	normal endometrium	increased	mRNA; proliferative phase vs. other phases	[10]
**PMCA1**	myometrium	increased	WB; labour vs. non-labour	[80]
**PMCA4**	myometrium	increased	mRNA; labour vs. non-labour	[81]
**RyR**				
**RyR1**	myometrium	constant	mRNA; pregnancy	[82]
**RyR1**	uterine fibroids	increased	mRNA; WB vs. non-fibroids	[83]
**RyR2**	myometrium	increased	mRNA; pregnancy vs. nonpregnant	[84]
**RyR3**	myometrium	constant	mRNA	[82]
**IP_3_R**				
**IP_3_Rs**	myometrium	constant	IP_3_ binding assay; regardless of pregnancy status	[85]
**IP_3_R1**	uterine fibroids	increased	mRNA; WB; vs. adjacent myometrium	[83]
**MCU complex**				
**MCU**	placenta	increase	mRNA; WB; pregnancy vs. nonpregnant	[86]
**MCU**	endometrial cancer	increased	IHC; IF vs. normal endometrium	[87]
**MICU1**	placenta	increase	mRNA; WB; pregnancy vs. nonpregnant	[86]
**MCU**	myometrium	increase	mRNA; preterm birth vs. term birth	[86]
**MCUb**	myometrium	increase	mRNA; preterm birth vs. term birth	[86]
**EMRE**	myometrium	increase	mRNA; preterm birth vs. term birth	[86]
